# Cognitive impairment and neuropsychiatric symptoms among individuals with history of symptomatic SARS-CoV-2 infection: a retrospective longitudinal study

**DOI:** 10.1590/1980-5764-DN-2023-0053

**Published:** 2024-02-09

**Authors:** Nariana Mattos Figueiredo Sousa, Ana Claudia Paradella Freitas Maranhão, Lucia Willadino Braga

**Affiliations:** 1 Rede SARAH de Hospitais de Reabilitação, Salvador BA, Brazil.; 2 Rede SARAH de Hospitais de Reabilitação, Brasília DF, Brazil.

**Keywords:** COVID-19, SARS-CoV-2, Cognitive Dysfunction, Longitudinal Studies, COVID-19, SARS-CoV-2, Disfunção Cognitiva, Estudos Longitudinais

## Abstract

COVID-19 is a multisystem disease caused by the RNA virus (coronavirus 2 or SARS-CoV-2) that can impact cognitive measures.

**Objective:**

To identify the main cognitive and neuropsychiatric symptoms in adults who had no cognitive complaints prior to the infection. Specifically, to observe the trajectory of cognitive and neuropsychiatric performance after 6 months.

**Methods:**

This is a retrospective longitudinal study. Forty-nine patients (29 reassessed after 6 months), with a positive PCR test, with no prior cognitive complaints that only presented after the infection and without a history of structural, neurodegenerative or psychiatric neurological diseases. A brief cognitive assessment battery (MoCA), the Trail Making Test (TMT-A, B, ∆), and the Verbal Fluency Test were used, as well as the scales (Hospital Anxiety and Depression Scale-HADS, Fatigue Severity Scale-FSS). Correlation tests and group comparison were used for descriptive and inferential statistics. Level of significance of α=5%.

**Results:**

Mean age of 50.4 (11.3), 12.7 (2.8) years of education, higher percentage of women (69.8%). No psycho-emotional improvement (depression and anxiety) was observed between the evaluations, and patients maintained the subjective complaint of cognitive changes. The HAD-Anxiety scale showed a significant correlation with TMT-B errors. The subgroup participating in cognitive stimulation and psychoeducation showed improvement in the global cognition measure and the executive attention test.

**Conclusion:**

Our results corroborate other studies that found that cognitive dysfunctions in post-COVID-19 patients can persist for months after disease remission, as well as psycho-emotional symptoms, even in individuals with mild infection. Future studies, with an increase in casuistry and control samples, are necessary for greater evidence of these results.

## RESUMO.

A COVID-19 é uma doença multissistêmica causada pelo vírus RNA (coronavírus 2 ou SARS-CoV-2) que pode ocasionar repercussão em medidas cognitivas.

**Objetivo:**

Identificar os principais sintomas cognitivos e neuropsiquiátricos em adultos sem queixas cognitivas anteriores à infecção. Especificamente, verificar a trajetória do desempenho cognitivo e neuropsiquiátrico após 6 meses.

**Métodos:**

Trata-se de um estudo retrospectivo e longitudinal. Foram incluídos 49 pacientes (29 reavaliados após 6 meses), com exame de PCR positivo, sem queixas cognitivas prévias que só se apresentaram após a infecção ou histórico de doenças neurológicas estruturais ou neurodegenerativas. Foram utilizados a bateria de avaliação cognitiva breve (MoCA), o Teste de Trilhas (TMT-A, B e ∆) e o Teste de Fluência Verbal; assim como a Escala Hospitalar de Ansiedade e Depressão (HADS), e a escala de Severidade da Fadiga (ESF/BR). Testes de correlação e comparação de grupos foram utilizados para estatística descritiva e inferencial. Esta ocorreu através de. Nível de significância de α=5%.

**Resultados:**

idade média de 50,4 anos (11,3), anos de escolaridade 12,7 (2,8), maior proporção de mulheres (69,8%). Não foi observada melhora psicoemocional (depressão e ansiedade) entre as avaliações, assim como os pacientes mantiveram a queixa subjetiva de alteração cognitiva. A escala HAD-Ansiedade apresentou correlação significativa com os erros do TMT-B. O subgrupo que participou da estimulação cognitiva e psicoeducação apresentou melhora na medida de cognição global e no teste de atenção executiva.

**Conclusão:**

Nossos resultados corroboram com os demais estudos que constataram que a disfunção cognitiva em pacientes pós-COVID-19 pode persistir por meses após a remissão da doença, assim como sintomas psicoemocionais, mesmo em indivíduos com quadros leves da infecção. Estudos futuros, com aumento de casuística e amostras de controle, são necessários para maior evidência desses resultados.

## INTRODUCTION

COVID-19 is a multisystem illness caused by the RNA virus (coronavirus 2 or SARS-CoV-2). The virus connects to a receptor in the cell surface (ACE2), inducing its internalization and initiating the replication cycle. In viral infections, immune cells detect pathogenic RNAs and activate the inflammatory response, initiating wide-range effects that prevent the proliferation of the pathogen. However, SARS-CoV-2 can surpass this restraint, leading to a positive response among viral propagation. This mutual amplification causes a disordered increase in circulating inflammatory cytokines. The storm of inflammation, caused by SARS-CoV-2, is the main reason why the disease has long range physiological effects^1^ ^,^ ^2^.

Cognitive impairment is a frequent complication of COVID-19, and patients who have persistent symptoms after the initial SARS-CoV-2 infection are referred to as having Long COVID^3^ ^,^ ^4^ ^,^ ^5^. Factors associated with the disease (viral involvement and central nervous system dysfunction), social isolation and psycho-emotional disorders, as well as their treatment, may contribute to the emergence of cognitive sequelae in these individuals^6^.

Crivelli et al. also reported that cognitive symptoms may occur in patients after recovery from COVID-19, regardless of disease severity, and can last for months after remission. Therefore, cognitive assessment should be included in post-COVID-19 follow-up, being emphasized for the design of cognitive rehabilitation programs^7^.

Post-COVID-19 cognitive complaints can persist after 12 months of follow-up, but mainly in women and patients with increased antinuclear antibodies^8^. A British study showed that high levels of C-reactive protein, for 7 months or more, may be related to the persistence of cognitive impairment^9^. While none of these studies can confirm the prognosis of prolonged cognitive impairment related to COVID-19, the initial inflammatory response can persist for several months after the acute infection, disrupting the immune response and leading to cognitive symptoms, which may improve as the inflammation subsides^10^ ^,^ ^11^.

However, these cognitive changes may be reversible or transient. According to Del Brutto et al., six months after infection, only COVID-19 survivors had a significant decline in MoCA scores (β=-1.37, 95%CI -2.14 to -0.61, p<0.001), which was reversed after 1 (one) additional year of follow-up (β=0.66, 95%CI -0.11 to 1.42, p=0.092). No differences were observed between uninfected individuals when the two post-pandemic MoCA battery scores were compared^12^. Improvement in MoCA battery (but less than baseline) was identified after 18 months of follow-up in mild post-COVID-19 subjects when compared to subjects without virus infection. Brazhenets et al. also observed improvement in hypometabolism (PET-FGD) and cognition (MoCA) from the subacute stage to the chronic stage^13^.

A study conducted by Braga et al. evidenced that, regardless of the severity of COVID-19 syndrome, patients have problems related to their executive function and a high incidence of anxiety and depression, reinforcing the need for neurorehabilitation programs and data generation for public health measures. In this study, 614 adults participated and were evaluated, on average, eight months after infection. Participants were, on average, 47.6 years old, seeking rehabilitation for neuropsychological problems. Patients were assessed using the Barrow Neurological Institute Screen for Higher Cerebral Functions (BNIS), Phonemic Verbal Fluency and Clock Drawing (NEUPSILIN) tests for executive functions, and the Hospital Anxiety and Depression Scale (HADS)^14^.

For the intervention in cognitive measures, studies indicate the use of strategies already consolidated in other neurological and acquired conditions (consensus-based recommendations), such as training or cognitive stimulation and implementation of compensatory strategies. This way, psychoeducation would be a relevant tool for relieving anguish and suffering in this population. Thus, individualized assessment allows referring the patient to a rehabilitation program based on their needs, reducing morbidity and improving their quality of life^15^ ^,^ ^16^.

Regarding neuropsychiatric disorders and COVID-19, there is a basis in the literature on the relationship between psychological stress, decreased neuroplasticity and increased neuroinflammatory processes, such as increased inflammatory cytokines and neurotoxic effects^17^ ^,^ ^18^. Likewise, psycho-emotional disorders such as depression and anxiety also have these neurobiological substrates^19^.

In addition to pandemic-related psychological stress, other biological mechanisms have been studied to allow understanding of the neuropsychiatric symptoms seen in patients with COVID-19 (inflammation, respiratory disease, and even changes in the opioid system).

Neuropsychiatric symptoms in COVID-19 can also persist months after hospitalization, mainly in patients who required intensive care for severe disease^2^ ^,^ ^20^. The study by Almeria et al. associated anxiety and depression with cognitive complaints^21^.

Patients who had COVID-19 appear to be at greater risk of psychiatric sequelae, and a psychiatric diagnosis can be an independent risk factor for COVID-19. Cohort studies are required^22^.

Therefore, the main objective of this study was to identify the major cognitive and neuropsychiatric symptoms in adults without cognitive complaints prior to infection. Specifically:

- to describe the sociodemographic (age, sex, education) and clinical profile (neurological manifestations, hospitalization, and number of days, invasive mechanical ventilation (IMV), delirium, sleep quality, fatigue), and psychosocial aspects (depression, anxiety) in COVID-19 patients;
- to characterize the patients’ cognitive complaints and correlate cognitive data with clinical data (time of exposure to infection, hospitalization, invasive ventilatory support, delirium, previous psychiatric diseases, and neurological manifestations);
- to verify the evolution of cognitive and neuropsychiatric performance after 6 months in patients; and
- explore whether intervention strategies focused on cognition contribute to these results.

## METHODS

### Design of study

This is a retrospective, descriptive and observational longitudinal study.

### Participants and data collection

**Figure 1.**
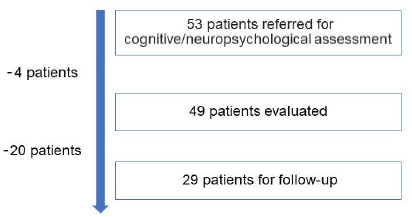
Flow-chart for recruitment of patients in post-COVID-19 syndrome cognition assessments.

From June 2021 to November 2022, 53 patients were referred for cognitive/neuropsychological evaluation, after infection with SARS-CoV2, confirmed by a positive polymerase chain reaction (PCR) test to detect viral RNA, from Rede SARAH de Hospitais de Reabilitação (Salvador, BA, Brazil). These patients were referred to assessment after cognitive complaints in the admission consultation with the medical team in the above-mentioned Hospital. The following inclusion criteria were used:

- patients without prior cognitive complaints, reported spontaneously by the patient during the structured clinical interview, identified through the retroactive search in electronic medical records, which were only presented after infection (memory, attention, slowed thinking, language, organization, planning, among others);
- patients without a history of structural, neurodegenerative or psychiatric neurological diseases;
- patients with complete assessment protocols. The final number of participants was 49 patients. After 6 months, 29 of these patients underwent cognitive and psycho-emotional reassessment (Figure 1), with 19 patients (65.5%) participating in the cognitive intervention group, and 10 patients (34.4%) not participating or not completing the number of protocol sessions.

**Figure 2.**
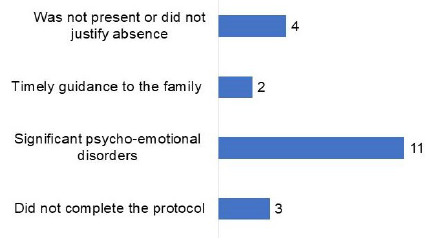
Factors related to not undergoing cognitive intervention.

It should be noted that 11 (22.4%) of these 49 patients showed psycho-emotional worsening (mood and/or anxiety disorder), with time not being appropriate for cognitive evaluation or intervention; 3 (6.1%) did not complete the protocol; 2 (4.0%) received timely guidance to the family; and 4 (8.1%) were not present or did not justify absence (Figure 2). These patients received psychological support and were referred for psychotherapeutic and psychiatric follow-up, in addition to receiving guidance on non-pharmacological management strategies.

The evaluation in the intervention group was conducted by 2 (two) specialists in cognition, once a week, for 90 minutes, totaling 4 sessions (360 minutes). The intervention consisted of paper-and-pencil tasks, organized at a level of complexity, and targeted at specific cognitive domain(s) aiming at improving cognitive function, such as attention, executive functions and memory. The intervention sessions were aimed at improving the functional task, that is, gaining independence and autonomy. Psychoeducation was also used, with explanations about cognitive impairment and compensatory strategies to aid functionality.

The participants were from the neurological rehabilitation program, referred for psychological care after reporting persistent cognitive complaints following a medical admission consultation. Exclusion criteria included significant impairment of the upper limbs, changes in visual acuity or the visual field that could interfere with assessment, drug use, or psychiatric disorders. This study was approved by the local Research Ethics Committee (CAAE: 56800322.7.0000.0022).

### Cognitive assessment

Data from cognitive assessments were included (baseline and after 6 months) in electronic medical records. The instruments used were Montreal Cognitive Assessment (MoCA), Trail Making Test (TMT) A, B, ∆ (B-A), and Verbal Fluency Test.

### Questionnaires

The Hospital Depression and Anxiety Scale (HADS) and the Fatigue Severity Scale (FSS) were used. Other clinical data were also gathered from the electronic medical records: time and severity of infection, hospitalization, invasive mechanical ventilation, neurological injury, sleep disturbance, arterial hypertension, diabetes, lung disease, delirium and changes in sensory perception and functionality.

### Data analysis

The participants’ characteristics were described by their mean and standard deviation in the case of continuous variables and by their absolute frequency and percentage in the case of categorical (nominal) variables. Some of these data were presented in the form of bar diagrams. Differences between participants in the two evaluations (baseline and after 6 months) were analyzed using the non-parametric Wilcoxon test. Differences between groups were assessed using one-way ANOVA and the Mann-Whitney U test according to data distribution, as well as analysis of the correlation between clinical and cognitive variables through a non-parametric test (Spearman rank correlation).

The Shapiro-Wilk and Kolmogorov-Smirnov tests were used to confirm (or rule out) the normal distribution of the data sets.

The SPSS statistical software (version 25.0) was used for data analysis.

The significance level was set at p<0.05.

## RESULTS

A total of 49 individuals were included in this study, out of which 29 were reassessed after 6 (six) months. The sample consisted of an average age of 50.4 (11.3), 12.7 (2.8) years of education, higher percentage of women (69.8%). Regarding clinical data, the interval between SARS-CoV-2 infection and the clinical evaluation was 9.08 (5.00) months; 39.6% of the patients evaluated were hospitalized, 18.8% were put on invasive mechanical ventilation, 11.3% had neurological damage, 9.4% had delirium, 50.9% had sleep disorders, 58.4% had hypertension, 26.4% had diabetes, 7.5% had lung disease, and 13.2% had changes in sensory perception. All patients with neurological sequelae were included in the analysis and underwent medical evaluation, imaging and laboratory tests.

Two patients had brain damage resulting from critically ill encephalopathy (MRI with multiple microhemorrhagic foci); 2 ischemic stroke (1 case underwent MRI in another hospital, report not available; the other case computed tomography reported sequelae of vascular events in the posterosuperior aspect of the right frontal lobe, in ipsilateral temporooccipital topography and in the left frontal lobe, suggestive of embolic source); 1 hemorrhagic stroke (parenchymal lesion with previous hemorrhagic component of corticosubcortical predominance extending to the corona radiata in the right parietal region); 1 post-meningoencephalitis hydrocephalus (we do not have data on the etiology of meningoencephalitis, MRI with ventricular shunt catheter, hypersignal in cerebellar deep white matter, periventricular of the fourth ventricle, periaqueductal region, subependymal regions of the third and lateral ventricles, chiasm/optic tract, gyrus rectus/olfactory bulb on the right, without intravenous contrast enhancement, related sequelae of the previous intraventricular inflammatory/infectious process) (Table 1).

**Table 1.** Demographic and clinical characteristics of the sample.

n=49	Mean (SD) or n (%)
Age, years	50.43 (11.3)
Sex	
Female	37 (69.8)
Male	16 (30.1)
Education, years	12.79 (2.8)
Hand preference	
Right-handed	47 (88.6)
Left-handed	5 (9.4)
Ambidextrous	1 (1.8)
COVID-19 infection, months	9.08 (5.0)
Hospitalization	21 (39.6)
Invasive mechanical ventilation	10 (18.8)
Fatigue	17.02 (24.1)
Neurological damage	6 (11.3)
Delirium	5 (9.4)
Sleep disorder	27 (50.9)
Hypertension	31 (58.4)
Diabetes	14 (26.4)
Pulmonary disease	4 (7.5)
Disorder of sensory perception	7 (13.2)

Of the patients who performed below the cutoff score on the MoCA test at baseline, 20 (40.8%) had mild to moderate impairment (MoCA 18-25), and 9 (18.3%) had severe impairment (MoCA 10-17). After six months, 12 (41.3%) had mild to moderate impairment (MoCA 18-25) and 5 (17.4%) had severe impairment (MoCA 10-17). Therefore, there was no statistically significant difference between the two evaluations. This result was also observed for those patients with scores greater than or equal to 26, that is, at baseline 18 patients (36.7%) and, after 6 months, 11 patients (37.9%).

As for neuropsychological tests, data showed that there was a statistically significant difference between the subgroups (initially and after 6 months) in the Trail Making Test-B errors (p=0.029), and the semantic verbal fluency test (p=0.058). Despite the reduction in the Hospital Depression and Anxiety Scale (HAD) score, no improvement in depression and anxiety symptoms was observed. It should be noted that these patients maintained the subjective complaint of cognitive changes, which was emphasized, but was less detailed (lower percentage) when compared to the initial/baseline assessment (Table 2).

**Table 2.** Cognitive and neuropsychological data (baseline and after 6 months).

	Mean (SD) or n (%) baseline n=49	Mean (SD) or n (%) after 6 months n=29	p-value
MoCA			
Global score	22.0 (5.8)	22.8 (6.2)	0.094
<26	31.0 (63.2)	18.0 (62.0)	0.916
18-25	20.0 (40.8)	12.0 (41.3)	0.961
10-17	9.0 (18.3)	5.0 (17.2)	0.900
≥26	18.0 (36.7)	11.0 (37.9)	0.916
TMT			
A sec	96.8 (74.6)	89.9 (53.1)	0.854
A errors	0.7 (1.0)	7.5 (38.5)	0.106
B sec	174.5 (104.1)	160.6 (67.1)	0.163
B errors	2.0 (1.7)	1.7 (1.8)	0.029*
(B-A)	77.7 (51.3)	70.7 (40.7)	0.225
Verbal fluency			
Categorical (animals)	15.2 (6.5)	16.4 (7.1)	0.058
HAD			
Depression	9.3 (5.3)	7.6 (4.2)	0.296
Anxiety	9.7 (4.6)	9.6 (5.0)	0.868
Cognitive complaints			
Emphasized	93.5%	88.6%	-
Detailed	88.7%	76.3%	-

Abbreviations: MoCA, Montreal Cognitive Assessment; TMT, Trail Making Test; HAD, Hospital Depression and Anxiety Scale.

Note: Wilcoxon test (Wilcoxon matched-pairs or Wilcoxon signed-rank test). *Significance level <0.05.

When comparing cognitive and neuropsychiatric data between patients who participated (n=19) and did not participate in the cognitive intervention + psychoeducation (n=10), the subgroup of patients who participated in this non-pharmacological approach presented better cognitive scores after 6 months, that is, there was a statistically significant difference in the global assessment battery of cognition and more executive attention test - Trail Making Test-B errors (Table 3).

**Table 3.** Cognitive and neuropsychiatric data (baseline and after 6 months).

Cognitive intervention	Mean (SD) baseline	Mean (SD) after 6 months	p-value
**Yes (n=19)**			
MoCA			
Global score	22.1 (6.0)	23.3 (5.3)	0.022*
<26	18.7 (5.0)	21.4 (5.2)	0.019*
18-25	21.8 (1.6)	24.4 (2.5)	0.021*
10-17	14.0 (2.9)	16.0 (4.2)	0.842
≥26	27.9 (1.3)	27.8 (2.3)	0.579
TMT			
A sec	99.6 (83.4)	86.4 (49.4)	0.804
A errors	0.7 (1.1)	16.3 (57.5)	0.393
B sec	175.2 (116.1)	164.0 (72.4)	0.412
B errors	1.9 (1.6)	1.4 (1.8)	0.046*
(B-A)	75.5 (45.2)	77.6 (49.8)	0.262
Verbal fluency			
Categorical (animals)	14.6 (5.4)	15.7 (6.9)	0.060
HAD			
Depression	9.2 (4.8)	7.6 (3.7)	0.366
Anxiety	10.0 (5.2)	9.3 (5.1)	0.262
**No (n=10)**			
MoCA			
Global score	22.0 (5.7)	22.2 (7.4)	0.915
<26	19.0 (5.0)	18.1 (8.1)	1.000
18-25	21.8 (2.8)	22.0 (4.7)	0.396
10-17	15.8 (1.26)	13.0 (-)	0.317
≥26	27.4 (1.4)	27.0 (1.7)	0.831
TMT			
A sec	94.4 (68.1)	92.6 (57.3)	0.917
A errors	0.7 (1.1)	0.4 (1.0)	0.152
B sec	173.9 (95.5)	157.8 (64.8)	0.235
B errors	2.1 (1.7)	2.0 (1.8)	0.271
(B-A)	79.5 (56.7)	65.1 (32.3)	0.452
Verbal fluency			
Categorical (animals)	16.0 (7.7)	17.2 (7.5)	0.417
HAD			
Depression	9.3 (5.6)	7.5 (4.8)	0.587
Anxiety	9.5 (4.4)	9.8 (5.1)	0.526

Abbreviations: MoCA, Montreal Cognitive Assessment; TMT, Trail Making Test; HAD, Hospital Depression and Anxiety Scale.

Notes: Wilcoxon test (Wilcoxon matched-pairs or Wilcoxon signed-rank test). *Significance level <0.05.

A statistically significant correlation was observed, at baseline, between the time of infection by the SARS-CoV-2 and cognitive measures (MoCA and Trail Making Test-B errors, Trail Making Test-∆), hospitalization and cognitive measures (MoCA and Trail Making Test-A-second, Trail Making Test-B-second Test, Trail Making Test-∆ and Verbal Fluency), invasive mechanical ventilation and cognitive measures (MoCA, Trail Making Test-B-second and Verbal Fluency), diabetes and cognitive measures (Trail Making Test-B-second, Trail Making Test-A-errors, Trail Making Test-B-errors and Trail Making Test-∆), lung disease and cognitive measurement (Trail Making Test-B-errors) and altered sensory perception and cognitive measurement (Verbal Fluency).

After 6 months there was a statistically significant correlation between hospitalization and global cognitive measurement (MoCA), invasive mechanical ventilation and MoCA, diabetes and attentional measurement (Trail Making Test-A second). Thus, less association was observed between cognitive variables and clinical data (Table 4).

**Table 4.** Correlation (p) between clinical and cognitive data.

	Infection COVID-19 (a)	Hospitalization (c)	Invasive mechanicalventilation (c)	Delirium (c)	Sleepdisorder (c)	Hypertension (c)	Diabetes (c)	Pulmonarydisease (c)	Change of sensory perception (c)
**Baseline**									
MoCA									
Global score	-0.378 (0.008)*	0.058	0.013*	0.545	0.446	0.703	0.062	0.133	0.062
<26	-0.115 (0.544)	0.031*	0.093	0.098	0.359	0.648	0.329	0.767	0.118
18-25	0.004 (0.987)	0.502	0.591	-	0.144	0.171	0.314	0.484	0.378
10-17	-0.151 (0.698)	0.291	0.532	0.166	0.018	0.510	0.429	-	0.104
≥26	-0.532 (0.023)*	0.287	0.620	0.193	0.201	0.110	0.059	-	0.620
TMT									
A sec	-0.014 (0.922)	0.111	0.187	0.647	0.535	0.837	0.347	0.306	0.076
B sec	0.268 (0.066)	0.011*	0.016*	0.794	0.760	0.754	0.039*	0.315	0.140
A errors (b)	0.687	0.517	0.679	1.000	0.634	0.421	0.053	0.838	0.125
B errors (b)	0.025*	0.712	0.642	1.000	0.494	0.676	0.041*	0.049*	0.182
(B-A)	0.297 (0.040)*	0.004*	0.095	0.571	0.810	0.579	0.026*	0.476	0.902
Verbal fluency									
Categorical (animals)	-0.245 (0.094)	0.017*	0.012*	0.389	0.841	0.188	0.074	0.700	0.012*
**After 6 months**									
MoCA									
Global score	-0.066 (0.735)	0.027*	0.013*	0.462	0.786	0.327	0.230	0.046*	0.589
<26	0.085 (0.738)	0.120	0.020*	0.099	0.893	0.649	0.929	0.090	0.777
18-25	0.305 (0.335)	0.566	0.129	-	0.467	0.122	0.627	0.107	1.000
10-17	0.158 (0.800)	0.139	0.374	-	0.554	0.554	0.374	-	0.717
≥26	0.420 (0.199)	0.055	0.749	0.749	0.214	0.781	0.633	-	0.749
TMT									
A sec	0.025 (0.896)	0.135	0.235	0.829	0.946	0.411	0.043*	-	0.566
B sec	0.175 (0.365)	0.220	0.106	0.630	1.000	0.636	0.089	-	0.662
A errors (b)	0.621	1.000	0.362	0.207	0.617	0.260	0.404	0.037*	0.515
B errors (b)	0.773	0.608	0.198	1.000	0.510	0.205	0.415	0.365	0.801
(B-A)	0.172 (0.373)	0.582	0.388	0.698	0.636	0.491	0.550	-	0.914
Verbal fluency									
Categorical (animals)	-0.083 (0.669)	0.226	0.137	0.574	0.573	0.755	0.153	0.154	0.719

Abbreviations: MoCA, Montreal Cognitive Assessment; TMT, Trail Making Test.

Notes: (a) Spearman’s rho (coefficient and p-values) for COVID-19 Infection (months), except Trail Making Test errors - p from analysis of variance (ANOVA); (b) Fisher’s exact test p-values for Trail Making Test errors; (c) Mann-Whitney test p-values for the other variables. *Significance level <0.05.

The level of education correlated with the most cognitive measures (baseline and after 6 months), suggesting that schooling can interfere with cognition after the COVID-19 condition.

The HAD-Anxiety scale showed a significant correlation with Trail Making Test-B errors. After 6 months, no statistically significant correlation was observed between these variables, except for education (Table 5).

**Table 5.** Correlation (p) between Hospital Depression and Anxiety Scale and cognitive data.

	HAD-Depression	HAD-Anxiety	Age	Education
**Baseline**				
MoCA				
Global score	0.041 (0.804)	-0.183 (0.269)	-0.028 (0.848)	0.548 (0.000*)
<26	0.253 (0.255)	-0.066 (0.771)	-0.181 (0.330)	0.344 (0.058)
18-25	0.441 (0.087)	0.092 (0.735)	-0.191 (0.420)	0.411 (0.072)
10-17	0.316 (0.684)	0.400 (0.600)	-0.428 (0.144)	-0.183 (0.638)
≥26	-0.364 (0.166)	-0.049 (0.858)	0.064 (0.800)	-0.011 (0.965)
TMT				
A sec	-0.090 (0.589)	0.165 (0.320)	0.037(0.799)	-0.452 (0.001*)
B sec	-0.050 (0.761)	0.259 (0.115)	0.004 (0.977)	-0.523 (0.000*)
A errors	0.413	0.941	0.168	0.051
B errors	(0.570)	(0.020)	(0.828)	(0.001*)
(B-A)	0.129 (0.437)	0.159 (0.337)	-0.007 (0.958)	-0.396 (0.004*)
Verbal fluency				
Categorical (animals)	-0.144 (0.386)	-0.258 (0.117)	-0.140 (0.335)	0.512 (0.000*)
**After 6 months**				
MoCA				
Global score	-0.280 (0.140)	-0195 (0.310)	-0.232 (0.224)	0.623 (0.000*)
<26	-0.213 (0.397)	-0.096 (0.706)	-0.220 (0.381)	0.413 (0.089)
18-25	0.068 (0.834)	0.195 (0.544)	-0.581 (0.048*)	0.682 (0.015*)
10-17	-0.462 (0.434)	-0.553 (0.334)	-0.763 (0.133)	0.895 (0.040*)
≥26	-0.216 (0.523)	0.030 (0.929)	-0.254 (0.451)	0.361 (0.275)
TMT				
A sec	0.198 (0.301)	0.154 (0.424)	0.208 (0.278)	-0.608 (0.000*)
B sec	0.196 (0.306)	0.135 (0.484)	0.128 (0.506)	-0.586 (0.000*)
A errors	0.407	0.173	0.460	0.016
B errors	(0.986)	(0.399)	(0.471)	(0.009*)
(B-A)	0.223 (0.244)	0.088 (0.646)	0.018 (0.924)	-0.084 (0.663)
Verbal fluency				
Categorical (animals)	-0.042 (0.826)	-0.072 (0.709)	-0.237 (0214)	0.632 (0.000*)

Abbreviations: MoCA, Montreal Cognitive Assessment; TMT, Trail Making Test.

Notes: ANOVA p-values for Trail Making Test, errors; Spearman’s rho (coefficient and p-values) for the other variables; *Significance level <0.05.

## DISCUSSION

In this study, we evaluated the presence of cognitive impairment in patients with COVID-19 with complaints that persisted after the acute phase. According to data from the literature, the progression of neuropsychological changes after COVID-19 infection over time has not yet been elucidated. Persistence of neuropsychological disturbances, impaired quality of life, anxiety and depressed mood, sleep disturbances and fatigue have been described in up to 23% at a 3-month follow-up in a cohort of patients who recovered from COVID-19^23^.

A relevant fact of this study is that the sample consisted of a higher proportion of women. This higher number of female patients suggests that long COVID seems to be related to some sex-dependent factors. The expression of angiotensin-converting enzyme 2 (ACE-2) and serine transmembrane protease 2 (TMPRSS2), which are important targets for the virus to access cells, may explain the unfavorable outcome in the acute phase, which is more common in men than in women. Geneticfactors related to the immune response in women, such as greater IFN1 activity, greater expression of TRL7 (a common virus sensor that is located on the X chromosome) and expression of sex hormones, are factors that may explain a possible cause for better inflammatory response in women. Another hypothesis is that, in the long COVID syndrome, traits of SARS-COV-2 remain in various organs, such as the kidneys, heart, liver, gastrointestinal tract, and brain. In the brain, they can activate an inflammatory cascade, interfering with the activity of the central nervous system, leading to “brain fog”^24^. Furthermore, we could hypothesize that women are, in general, more attentive to their bodies and related changes, and they seek health services more than men.

In the present study, we observed that most patients with cognitive complaints had insomnia, depression, and anxiety. These patients persisted with symptoms and cognitive complaints after the intervention. In a review article, female individuals were more likely to have the long COVID syndrome and the psychiatric subcategories than males, placing the female sex as a predictor of chronic fatigue and symptoms of behavioral disorders^25^. In a meta-analysis, 58% of post-COVID-19 patients reported a worsening of their quality of life and, of these, 37.5% had anxiety/depression^26^. Some viral infections can generate hippocampus atrophy, resulting in cognitive changes, memory loss and emotional dysregulation; thus, the neuropathological changes of SARS-CoV-2 may be associated to hippocampal abnormalities^27^. The mechanisms behind these symptoms are currently an area of investigation.

The vast majority of participants in this study scored below expectations on the MoCA total score (<26 points), as well as measures of executive attention (Trail Making Test-TMT and Verbal Fluency Test) when we take these patients’ average education (16 years) into account. Despite improvement after 6 months, they maintained the complaint of cognitive impairment, as well as symptoms of anxiety and mood disorders. In the study by Del Brutto et al., an improvement in the global measure of cognition was also observed, suggesting that prolonged cognitive decline related to COVID-19 may spontaneously improve over time, but remain lower than the pre-infection assessment levels^28^.

This study, therefore, points out the high frequency of cognitive dysfunction after COVID-19 infection. Data suggest that cognitive symptoms may occur in patients after recovery and can last for months after disease remission, even in cases of milder infections, making it important to include cognitive or neuropsychological assessment in post-COVID-19 protocols.

It is necessary to monitor these patients, as there is suffering, and psycho-emotional conditions associated with cognitive impairment. As reported by Badenoch et al., neuropsychiatric symptoms are common and persistent after recovery from COVID-19^29^ and may be associated with a worse cognitive performance^30^.

Despite the difference in the sample size between the two subgroups (baseline and after 6 months), the data indicate the persistence of anxiety and depression, but also improvement in measures of verbal fluency and, mainly, executive attention (divided and alternating attention), identified in the Trail Making Test B-errors. These cognitive alterations (verbal fluency and attention domains) are the most reported in the acute phase of the disease and up to 6 months after infection^5^ ^,^ ^31^ ^,^ ^32^, and this result may indicate that the COVID-19-related cognitive dysfunction may spontaneously improve over time.

Another aspect to be considered in this study is the significant correlation between the HAD-Anxiety scale and the Trail Making Test-B errors, that is, treatment strategies for psycho-emotional disorders are necessary to modulate the symptoms and, consequently, contribute to the cognitive complaints brought by patients.

Non-pharmacological intervention strategies targeting cognition are important in the cognitive trajectory, since we observed better performance in measures of global cognition and executive attention after participating in stimulation and psychoeducation groups, despite the reduced sample size of this study. As mentioned by Hagen et al., , the management of cognitive symptoms is necessary to improve the quality of life and functionality of patients^15^.

Thus, this study brought relevant data on the follow-up of post-COVID-19 patients, corroborating the literature on the persistence of cognitive impairment even in mild cases, but also improvement when compared to baseline assessment. This study also showed the persistence of cognitive complaints (a detailed and emphasized characteristic). Neuropsychiatric aspects are frequent and there was no change between assessments, besides being associated with cognitive measures, mainly anxiety, which may also explain why complaints are more emphasized and have a greater functional impact.

The limitations of this study include the retrospective design and small sample size, especially when stratifying the group after 6 (six) months (patients who participated and did not partake of the cognitive intervention), the lack of a control group and a higher proportion of patients with mild infection, in addition to the absence of an extensive neuropsychological assessment before and after the infection, which would allow a more accurate estimate of the impact of SARS-CoV-2 in cognitive functions. The patients included in this study requested a medical consultation, which can result in a population with heightened anxiety symptoms with the potential to interfere with cognitive performance.

In conclusion, this study aimed to identify the cognitive and behavioral profile of post-COVID-19 patients, as well as to evaluate the persistence of the complaint and its trajectory after 6 months.

The results showed that cognitive symptoms can persist after disease remission, as highlighted by previous studies. The persistence of symptoms of mood swings and anxiety, along with the maintenance of the subjective cognitive complaints in these individuals even in mild cases of the infection, are highlighted, reinforcing the importance of evaluation and referral to interdisciplinary rehabilitation programs.

Cognitive complaints in long COVID-19 were a frequent complication of COVID-19, and were diagnosed more in women than in men and in patients who had recovered from a mild form of the disease. Cognitive intervention (stimulation and psychoeducation) contributes to the improvement of this dysfunction, and this type of treatment is important concomitantly with psychological and psychiatric support. Long-term follow-up and future controlled studies are necessary for strengthening the statistical power and robustness of these results.
